# Variability in nutrient composition of cereal grains from different origins

**DOI:** 10.1186/s40064-016-2046-3

**Published:** 2016-04-06

**Authors:** Jinyoung Lee, Doo Seok Nam, Changsu Kong

**Affiliations:** Department of Animal Science and Technology, Konkuk University, Seoul, 05029 Republic of Korea

**Keywords:** Barley, Corn, Location, Variation, Wheat

## Abstract

Nutrient composition of individual feed ingredient in mixed feed is important for accurate formulation of animal feeds. However, each feed ingredient can be different depending on its origin. Therefore, this study was conducted to investigate the variability in nutrient compositions of corn, wheat, and barley grains from different origins. Cereal grains used in this study were from 5 countries for 432 corn samples, 5 countries for 65 wheat samples, and 3 countries for 60 barley samples. They were imported to Korea between 2006 and 2015. These grain samples were subjected to analysis for moisture, crude protein (CP), ether extract (EE), crude fiber (CF), ash, calcium (Ca), phosphorus (P), and gross energy (GE). The concentrations of moisture, CP, EE, CF, ash, Ca, P, and GE of corn differed (*P* < 0.05) among countries. GE in corn samples ranged from 3836 kcal/kg (Ukraine) to 3995 kcal/kg (Brazil). There were also differences (*P* < 0.05) in moisture, CP, ash, and P of wheat and in moisture, CF, Ca, P, and GE in barley from different countries. GE values in wheat ranged from 3957 kcal/kg (Brazil) to 4058 kcal/kg (United States) and GE values in barley samples ranged from 3894 kcal/kg (India) to 4059 kcal/kg (Australia). The most different nutrient depending on origins was Ca. The coefficient of variation was 65.7 % for corn, 57.4 % for wheat, and 28.8 % for barley. In conclusion, nutrients and energy contents in corn, wheat, and barley from various origins investigated in the present study were different. Therefore, it is important to consider these variations when formulating animal feeds.

## Background

The purpose of formulating animal diet is to improve the productivity by providing feed that meets nutrient requirements accurately. Therefore, it is important to formulate mixed diet through correct evaluating nutrient components in feed ingredients. According to a report by Korean authorities, annual feed production is about 19 million tons (Ministry of Agriculture, Food and Rural Affairs, MAFRA 2015). Most ingredients for animal feed is imported from overseas, with cereal grains usually accounting for approximately 60 % in animal feed as an energy source. Among cereal grains imported from other countries, corn is imported at the largest amount. Its nutrient compositions can vary due to many factors, including genetic and environmental factors (Watson and Ramstad 1987; Skogerson et al. 2010). In addition, wheat is widely-used in European countries. Nutrient contents of wheat can also vary due to several factors, including cultivars (Murphy et al. 2008) and environmental factors (Acharya and Sharma 1994). Barley is ranked the third among cereal grains used for feed in the United States. It is often used in human food and animal feed (Jadhav et al. 1998). Nutrient compositions of barley can also vary depending on genetic and environmental factors (MacGregor and Bhatty 1993; Jadhav et al. 1998). Because of these reasons, it is important to investigate nutrient values of cereal grains according to their origins so that feed formulation can be accurate. However, only a few studies have compared nutrient compositions in corn. No study has reported the variability in nutrient compositions of wheat and barley from different countries. Therefore, the objective of this study was to determine the variability in nutrient compositions of corn, wheat, and barley grains from different countries.

## Experimental procedures

### Ingredients data

The present study was conducted by using data from laboratories of major feed companies in Korea to understand the variations in nutrients of cereal grains imported to Korea from different countries. Corn and wheat nutrients data were from 2006 to 2015. Barley nutrients data were from 2010 to 2015. Corn grain was the main cereal grain imported to Korea. A total of 432 corn samples imported from 5 countries (Argentina, Brazil, China, Ukraine, and United States), 65 wheat samples from 5 countries (Australia, Brazil, India, Ukraine, and United States), and 60 barley samples from 3 countries (Australia, India, and Ukraine) were analyzed in this study.

### Chemical analysis

Cereal grain samples were analyzed for moisture, crude protein (CP), ether extract (EE), crude fiber (CF), ash, calcium (Ca), phosphorus (P), and gross energy (GE). All the chemical analyses were performed at the same laboratory with using same methodology for each nutrient and energy. Dry matter analysis was performed by drying in an oven at 135 °C for 2 h (method 930.15; AOAC, 2005). Analysis of CP was determined by using Dumas combustion method (Leco, St. Joseph, MI, USA). EE was analyzed after extracting crude fat with ether (method 920.39; AOAC, 2005). CF was analyzed using Ankom filter bag technique (Ankom technology, Macedon, NY, USA). Ash was determined with AOAC method 942.05. Ca and P were determined using inductively coupled plasma spectroscopy (method 985.91; AOAC, 2005). GE was determined with bomb calorimeter (IKA Calorimeter C 2000 basic; IKA-Werke GmbH, Staufen, Germany).

### Statistical analysis

Data were analyzed using GLM procedures of SAS (SAS Inst. Inc., Cary, NC, USA). The model consisted of country as an independent variable and nutrients as dependent variables as well as error term. The origin of country for all ingredients investigated had no interaction with year (data not shown). Therefore, pooled data averaged across year were used in the present study. In addition, the concentrations of nutrient in each country were not correlated to years (data not shown). An α-level of statistical significance was set at 0.05. Outliers that deviated 1.5 times of the interquartile ranges below the first quartile or above the third quartile were removed.

## Results and discussion

Although it is easier to compare nutrient values using “dry matter” basis than using “as-is” basis, every nutrient concentration was expressed in “as-is” basis in this study, because feed formulation is conducted at “as-is” basis in practice. Nutrient composition of feed ingredients in the swine NRC (2012) is widely used to compare nutritive values of ingredients. In addition, the values of NRC (2012) contain results of numerous studies. Therefore, they were used as references to discuss results of this study.

### Variation among countries

All nutrient concentrations evaluated for corn were different (*P* < 0.05) among countries (Table 1). The concentration of CP in corn ranged from 7.12 % from Brazil to 7.60 % from China. This range was slightly smaller than the range of 7.31–9.06 % reported by Cromwell et al. (1999) who investigated CP concentration of corn from various parts of the United States. This range was also smaller than the CP value of NRC (2012) at 8.24 % and that of CVB (2009) at 8.20 %. The EE content in corn varied from 3.30 % (United States) to 3.87 % (Brazil). The concentrations of Ca in corn from all the countries were fairly consistent to reported values at 0.02 % in NRC (2012) and CVB (2009) except a greater Ca content (0.04 %) in the corn from Ukraine. Phosphorus concentrations in corn ranged from 0.20 % (Brazil) to 0.23 % (Ukraine and United States), which were slightly lower compared to reported values (average of 0.26 %) by Cromwell et al. (1999). GE values in corn ranged from 3836 kcal/kg (Ukraine) to 3995 kcal/kg (Brazil). The GE contents in corn from this study were fairly consistent with values listed in NRC (2012). Brazil had relatively higher concentration of GE compared to other countries. This might be due to the relatively higher concentration of EE (3.87 %) in corn from Brazil. Ewan (1989) has suggested that the components in the prediction equation for GE are CP, EE, and ash and that GE is positively correlated to the concentration of EE. Many factors can affect the variation in nutrient components of corn, including cultivar (Feil et al. 2005; Ufaz and Galili 2008), fertilization (Kaiser et al. 2005), soil condition (Harder et al. 1982), and occurrence of toxins (Abbas et al. 2006). There have been efforts to improve nutrient composition of corn. Genetic approaches have been used to improve cultivars such as ‘quality protein maize’ with improved the concentration of lysine and tryptophan in seeds (Ufaz and Galili 2008). Therefore, CP content may vary depending on varieties. In addition, Feil et al. (2005) have found that there are significant differences in concentrations of CP, P, and Ca in grains of different genotypes. Harder et al. (1982) have shown that corn yield reduced up to 33 % depending on harshness and exposure of moisture stress that can affect nitrogen and consequently increase CP concentration in the corn. Rodrigues and Naehrer (2012) have reported that aflatoxin can be found in corn, soybean meal, wheat, and distillers dried grains with solubles from North and South America, Europe, and Asia from January 2009 to December 2011. It has been reported that aflatoxin in corn have been found under several stressful conditions such as drought, high temperatures, and the lack of nutrients in soil (Abbas et al. 2006). These conditions may decrease nutrient components in corn.

**Table 1:** Nutrient compositions of corn from different countries (as-is basis, %)

	Moisture	CP	EE	CF	Ash	Ca	P	GE
NRC (2012)								
n	133	163	115	78	76	61	76	48
Mean	11.69	8.24	3.48	1.98	1.30	0.02	0.26	3933
CV	20.62	11.29	22.41	30.81	24.62	50.0	19.23	2.19
CVB (2009)								
Mean	12.80	8.20	3.80	2.20	1.20	0.02	0.27	N/A
Argentina								
n	30	30	30	30	30	28	30	17
Mean	13.99	7.25	3.74	2.19	1.13	0.02	0.22	3869
CV	3.62	4.75	13.68	12.29	8.26	54.15	18.44	2.71
Brazil								
n	57	57	56	56	54	51	54	34
Mean	13.33	7.12	3.87	2.24	1.04	0.02	0.20	3995
CV	5.13	2.90	12.33	12.27	10.77	44.97	12.49	3.47
China								
n	56	56	55	56	56	48	56	56
Mean	14.39	7.60	3.62	2.27	1.14	0.02	0.21	3917
CV	4.17	2.86	8.44	16.92	10.29	73.55	14.37	1.12
Ukraine								
n	26	26	26	26	24	18	22	15
Mean	13.41	7.41	3.57	2.24	1.12	0.04	0.23	3836
CV	4.97	4.98	12.20	13.45	8.06	67.92	8.42	4.14
United States								
n	263	263	262	262	262	237	260	180
Mean	14.52	7.18	3.30	2.01	1.16	0.02	0.23	3902
CV	3.93	5.21	12.67	12.93	8.23	71.56	12.38	2.48
Maximum	14.52	7.60	3.87	2.27	1.16	0.04	0.23	3995
Minimum	13.33	7.12	3.30	2.01	1.04	0.02	0.20	3836
Average	13.93	7.31	3.62	2.19	1.12	0.02	0.22	3904
Among countries								
SEM	0.13	0.08	0.10	0.06	0.02	0.003	0.01	30.2
*P* value	<0.001	<0.001	<0.001	<0.001	<0.001	<0.001	<0.001	<0.001
CV	5.32	5.17	13.73	14.39	9.50	65.68	14.57	2.82

*CP* crude protein, *EE* ether extract, *CF* crude fiber, *Ca* calcium, *P* phosphorus, *GE* gross energy, *CV* coefficient of variation, *SEM* standard error of the mean

The concentrations of moisture, CP, ash, and P were different (*P* < 0.05; Table 2) in wheat samples from different countries. CP concentrations ranged from 10.55 % in wheat from Ukraine to 13.17 % in wheat from Brazil. Phosphorus concentration in wheat ranged from 0.26 % from Australia to 0.34 % from Brazil. Both CP and P values in the current study were closer to the values in CVB (2009) rather than to those of NRC (2012). The lowest GE was 3957 kcal/kg from Brazil and the highest GE was 4058 kcal/kg from United States. All GE values in this study were higher than that (3788 kcal/kg) listed in NRC (2012). Zijlstra et al. (1999) have investigated the mean GE of 16 wheat samples collected from Saskatchewan province of Canada. They reported that the GE was 4608 kcal/kg DM, which was fairly similar to the GE value of 4512 kcal/kg DM found in this study. In addition, the mean concentrations of CP, EE, CF, Ca, and P were consistent between the present study and the study of Zijlstra et al. (1999). Nutrient contents of wheat can vary depending on several factors such as cultivar (Murphy et al. 2008) and existence of toxins (Matthäus et al. 2004). Murphy et al. (2008) have indicated that mineral concentration in soft white wheat has decreased during the past 120 years, whereas the mineral concentration in hard red wheat has remained a constant concentration. It has been reported that wheat inoculated with *Fusarium culmorum* has significantly higher CP and ash contents than wheat without such inoculation (Matthäus et al. 2004).

**Table 2:** Nutrient compositions of wheat from different countries (as-is basis, %)

	Moisture	CP	EE	CF	Ash	Ca	P	GE
NRC (2012)^a^								
n	46	64	36	6	25	25	37	25
Mean	11.33	14.46	1.82	2.57	1.98	0.06	0.39	3788
CV	28.42	17.36	20.33	31.13	18.69	83.33	25.64	3.83
CVB (2009)								
Mean	13.20	11.10	1.30	2.40	1.50	0.04	0.31	N/A
Australia								
n	12	11	12	12	12	10	12	7
Mean	10.23	11.33	1.86	2.38	1.39	0.04	0.26	3982
CV	8.31	4.40	15.41	9.79	7.28	48.11	5.88	3.38
Brazil								
n	15	14	15	15	15	10	14	10
Mean	12.30	13.17	1.65	2.62	1.65	0.06	0.34	3957
CV	6.13	8.22	23.45	11.68	11.16	69.86	16.35	2.27
India								
n	12	13	13	13	12	11	13	7
Mean	10.91	11.70	1.74	2.42	1.61	0.07	0.30	4047
CV	4.80	3.78	17.82	8.94	7.64	31.16	12.25	3.59
Ukraine								
n	13	13	13	12	13	11	13	12
Mean	12.59	10.55	1.56	2.48	1.50	0.06	0.30	3980
CV	4.10	9.53	21.71	9.42	5.60	34.70	10.76	1.98
United States								
n	11	12	12	12	12	10	12	7
Mean	10.14	10.83	1.70	2.54	1.52	0.08	0.29	4058
CV	11.16	6.92	14.40	12.44	8.85	63.02	11.77	3.08
Maximum	12.59	13.17	1.86	2.62	1.65	0.08	0.34	4058
Minimum	10.14	10.55	1.56	2.38	1.39	0.04	0.26	3957
Average	11.23	11.52	1.70	2.49	1.54	0.06	0.30	4005
Among countries								
SEM	0.31	0.31	0.12	0.09	0.05	0.01	0.01	48.9
*P* value	<0.001	<0.001	0.256	0.074	<0.001	0.202	<0.001	0.115
CV	11.41	10.81	19.74	11.23	10.48	57.39	15.43	2.96

*CP* crude protein, *EE* ether extract, *CF* crude fiber, *Ca* calcium, *P* phosphorus, *GE* gross energy, *CV* coefficient of variation, *SEM* standard error of the mean

^a^Values of “Hard red wheat” were used

The concentrations of moisture, CF, Ca, P, and GE were different in barley samples from various countries (*P* < 0.05; Table 3). The concentrations of CP in barley samples from Australia and India were lower than those of NRC (2012) or CVB (2009). However, barley samples from Ukraine had similar CP concentrations to those of CVB (2009). The Ca and P contents in barley samples from various countries had very small differences. The concentrations of Ca in barley ranged from 0.05 % (Australia) to 0.07 % (Ukraine). Phosphorus values ranged from 0.25 % (India) to 0.28 % (Australia). The averaged Ca values were similar to those of NRC (2012) and CVB (2009). However, the concentrations of P in the present study (0.27 %) were lower than the reference value (0.35 %) of NRC (2012) or CVB (2009). Barley samples from Australia had slightly higher GE value (4059 kcal/kg) than those from other countries. Nutrient compositions of barley may vary depending on various factors, including cultivar (Gahoonia and Nielsen 1997), hulling process (Mitchall et al. 1976; Sumner et al. 1985), and weather (Leyshon and Sheard 1974). Gahoonia and Nielsen (1997) have suggested that barley cultivar with longer root hairs has better ability to absorb inorganic P from soil. It has been reported that hull-less barley has higher protein levels than covered barley (Mitchall et al. 1976; Sumner et al. 1985). However, the concentrations of indispensable amino acid in hull-less barley are not as high as the concentration of CP (Rhodes and Jenkins 1975; Sumner et al. 1985). In addition, Leyshon and Sheard (1974) have reported that the amount of nitrogen, P, and potassium in barley are decreased under short-term flooding condition.

**Table 3:** Nutrient compositions of barley from different countries (as-is basis, %)

	Moisture	CP	EE	CF	Ash	Ca	P	GE
NRC (2012)								
n	52	76	33	12	38	32	39	24
Mean	10.10	11.33	2.11	3.90	2.38	0.06	0.35	3939
CV	26.24	13.59	30.81	17.95	17.65	33.33	11.43	2.21
CVB (2009)								
Mean	13.10	10.40	1.70	4.60	2.10	0.06	0.35	N/A
Australia								
n	34	32	34	34	34	28	32	29
Mean	10.54	9.75	2.11	4.46	2.02	0.05	0.28	4059
CV	7.61	15.97	13.93	16.84	8.13	21.58	13.43	2.38
India								
n	17	19	19	16	18	19	18	17
Mean	10.26	9.46	2.14	5.81	2.10	0.06	0.25	3894
CV	9.36	4.59	25.67	3.91	7.14	26.05	11.17	2.72
Ukraine								
n	7	7	6	7	7	7	7	7
Mean	12.32	10.49	2.18	4.61	2.07	0.07	0.27	3918
CV	5.99	3.70	8.12	6.59	4.83	23.71	4.59	2.38
Maximum	12.32	10.49	2.18	5.81	2.10	0.07	0.28	4059
Minimum	10.26	9.46	2.11	4.46	2.02	0.05	0.25	3894
Average	11.04	9.90	2.15	4.96	2.06	0.06	0.27	3957
Among countries								
SEM	0.24	0.37	0.11	0.14	0.04	0.004	0.01	25.7
*P* value	<0.001	0.232	0.907	<0.001	0.159	<0.001	0.013	<0.001
CV	9.83	13.87	17.59	15.94	7.79	28.84	13.08	2.98

*CP* crude protein, *EE* ether extract, *CF* crude fiber, *Ca* calcium, *P* phosphorus, *GE* gross energy, *CV* coefficient of variation, *SEM* standard error of the mean

Coefficient of variation (CV, %) was used to estimate the variabilities in nutrient compositions. The concentration of Ca had relatively high CV in all ingredients because cereal grain contains very small amount of Ca. Therefore, a very small difference in Ca value could affect CV greatly. The CV values of corn in NRC (2012) for most nutrients except the Ca and GE were greater than those of the present study because NRC (2012) had much more nutrients data than this study. In addition, analyzed value of each nutrient might be different. Therefore, variation of nutrient in NRC (2012) could be greater than that of the current study. The CV values of GE in individual ingredients in this study were similar to those values of NRC (2012).

## Conclusion

Results of this study provided more detailed information about nutrient components of major cereal grains imported to Korea from various countries. There were some variations in nutrition contents depending on countries. Therefore, it is necessary to consider where these ingredients come from when formulating animal feeds in order to meet nutrient requirements accurately.
